# Do Balance Demands Induce Shifts in Visual Proprioception in Crawling Infants?

**DOI:** 10.3389/fpsyg.2019.01388

**Published:** 2019-06-20

**Authors:** David I. Anderson, Minxuan He, Paula Gutierrez, Ichiro Uchiyama, Joseph J. Campos

**Affiliations:** ^1^ Marian Wright Edelman Institute, San Francisco State University, San Francisco, CA, United States; ^2^ Department of Psychology, University of California, Berkeley, Berkeley, CA, United States; ^3^ Department of Psychology, Doshisha University, Kyoto, Japan

**Keywords:** balance, locomotion, motor development, optic flow, vision

## Abstract

The onset of hands-and-knees crawling during the latter half of the first year of life heralds pervasive changes in a range of psychological functions. Chief among these changes is a clear shift in visual proprioception, evident in the way infants use patterns of optic flow in the peripheral field of view to regulate their postural sway. This shift is thought to result from consistent exposure in the newly crawling infant to different patterns of optic flow in the central field of view and the periphery and the need to concurrently process information about self-movement, particularly postural sway, and the environmental layout during crawling. Researchers have hypothesized that the demands on the infant’s visual system to concurrently process information about self-movement and the environment press the infant to differentiate and functionalize peripheral optic flow for the control of balance during locomotion so that the central field of view is freed to engage in steering and monitoring the surface and potentially other tasks. In the current experiment, we tested whether belly crawling, a mode of locomotion that places negligible demands on the control of balance, leads to the same changes in the functional utilization of peripheral optic flow for the control of postural sway as hands-and-knees crawling. We hypothesized that hands-and-knees crawlers (*n* = 15) would show significantly higher postural responsiveness to movements of the side walls and ceiling of a moving room than same-aged pre-crawlers (*n* = 19) and belly crawlers (*n* = 15) with an equivalent amount of crawling experience. Planned comparisons confirmed the hypothesis. Visual-postural coupling in the hands-and-knees crawlers was significantly higher than in the belly crawlers and pre-crawlers. These findings suggest that the balance demands associated with hands-and-knees crawling may be an important contributor to the changes in visual proprioception that have been demonstrated in several experiments to follow hands-and-knees crawling experience. However, we also consider that belly crawling may have less potent effects on visual proprioception because it is an effortful and attention-demanding mode of locomotion, thus leaving less attentional capacity available to notice changing relations between the self and the environment.

## Introduction

One of the most pronounced and robust changes that follows the onset of hands-and-knees crawling is a shift in the way infants use vision to control posture. Hands-and-knees crawlers show greater postural responsiveness than pre-crawlers to perturbations of the visual surround that occur in the periphery of the field of view. This difference between hands-and-knees crawlers and pre-crawlers has been demonstrated in a “moving room” that permits independent movements of the front wall or the side walls and ceiling to perturb either the central or the peripheral visual surround (Higgins et al., 1996; Anderson et al., 2004; Lejeune et al., 2006; Uchiyama et al., 2008). Essentially, hands-and-knees crawlers show significantly greater synchronization between their postural sway and wall movements when the side walls and ceiling are suddenly moved toward them. The difference has also been demonstrated in a virtual moving room paradigm that uses moving images projected onto the walls of the room and measures the magnitude of infants’ postural sway (Ueno et al., 2018). In addition, significant increases in postural responsiveness to side-wall movement in a moving room have been induced in pre-crawling infants following 15 days of training in a powered-mobility-device (PMD) (Uchiyama et al., 2008; Dahl et al., 2013). In short, the shift in the way infants use vision to control posture following the onset of hands-and-knees crawling has been replicated several times, using different paradigms, and it has been induced by experimental manipulation of locomotor experience in a PMD. It is a robust phenomenon.

What accounts for the shift in the way infants use vision for postural control following hands-and-knees crawling onset? What demands does this type of locomotion make on the infant that would induce such a shift, and what specific experiences would facilitate the shift? The answers to these questions seem to lie in the roles vision plays in the control of hands-and-knees crawling and upright locomotion, roles that are quite different from those it plays in the control of stationary postures like sitting and standing. These roles were clearly articulated many years ago by Gibson (1979). Crucially, Gibson noted that when the head and eyes move through the environment, the visible surround literally flows across the retina. The flow patterns provide the basis for what Gibson (1979) referred to as *visual proprioception* – the awareness of self-movement that arises from the covariation between these flow patterns and the observer’s motion through the environment. The flow emanates from the destination to which the mover is headed during locomotion. The central retina is exposed to a radial optic flow pattern, like a starburst, and the peripheral retina is exposed to a lamellar optic flow pattern, like the lines of longitude on a globe, when the eyes are pointed toward the heading destination (Stoffregen, 1985). Though all areas of the retina show some sensitivity to the different flow geometries, it is exposure to lamellar flow in the periphery that induces the greatest sense of self-motion and postural compensation in standing young children and adults (Brandt et al., 1973; Berthoz et al., 1975; Lestienne et al., 1977; Stoffregen, 1985, 1986; Delorme and Martin, 1986; Stoffregen et al., 1987). Thus, adults and young children show the same postural responsiveness to peripheral lamellar optic flow (PLOF) as infants with hands-and-knees crawling experience.

What role does PLOF play in the control of locomotion? Just as in standing, PLOF plays a crucial role in the control of balance, an often overlooked subtask that must be managed during locomotion. Learning to maintain balance has been described as one of the most fundamental problems the child must master during the acquisition of the parade of motor skills that are learned during the early years of life (Adolph, 1997, 2002). Balance is a constraint on the organization and expression of all skilled activity, and this is particularly true for locomotor skills because of the variable magnitudes of the reactive forces on the whole body that result from changes in speed and changes in the terrain under the shifting bases of support during locomotion (Reed, 1989; Gibson and Pick, 2000). A prevailing idea is that if the hands-and-knees crawler can use PLOF to control balance, then central vision is freed to monitor the other, more intuitive, subtasks Gibson (1979) described as necessary during locomotion to a destination, namely, steering through a cluttered environment and ensuring the surface of support is traversable (Anderson et al., 2004).

Given the demands on the visual system during locomotion to concurrently monitor self-motion and the spatial layout, the new hands-and-knees crawler is likely pressed to differentiate spatially delimited patterns of optic flow so that steering, monitoring the surface, and maintaining balance can be done effectively, efficiently, and flexibly. Notably, infants show a fairly rapid increase in sensitivity to radial optic flow up to approximately 4 months of age, but that sensitivity changes little over the next few months and is still immature at 8 months of age (Gilmore et al., 2004, 2007; Shirai and Yamaguchi, 2010), the age at which many infants begin to crawl on hands and knees. Differentiation of perceptual information draws upon Gibson’s (1966) notion of “education of attention” and Gibson’s (1969) notion of “optimization of attention.” Such differentiation would be facilitated in the hands-and-knees crawler because of increased exposure to radial optic flow in the central field of view and lamellar optic flow in the periphery, particularly from the floor. Infants tend to look down at the floor and in the direction they are locomoting during crawling (Higgins et al., 1996; Kretch et al., 2014). The targets to which they locomote are often objects on the floor. If distracted by something in the periphery, they stop crawling and assume a side-sitting posture to examine the distraction (Higgins, 1994, unpublished dissertation; Soska et al., 2015).

However, infants must do more than just register and differentiate the information contained in the different optic flow patterns; they must also learn how to “functionalize” or utilize the new information for control of these tasks. Functionalization goes beyond differentiation in that it requires mapping of the information onto adaptive control strategies involved in accomplishing goals. In the current context, functionalizing PLOF indicates that the information contained in the optic flow patterns provides meaningful information about self-motion that can be utilized to control balance more effectively. A good example of the difference between discrimination and functionalization is the relation between binocular disparity and stereopsis. Experiments have demonstrated that infants between the ages of 5 and 10 weeks are capable of discriminating the disparity between the images projected on the retina of each eye (Appel and Campos, 1977; Seemiller et al., 2018); however, stereoscopic vision does not emerge until between 3 and a half and 6 months of age (Fox et al., 1980). Thus, the ability to discriminate binocular disparity does not automatically lead to functionalization of the information for stereopsis. Further development is required before binocular disparity information can be meaningfully utilized to perceive and track objects in three-dimensional space.

The background information presented thus far raises an interesting question. How would the acquisition of belly crawling, a mode of locomotion acquired by many infants prior to the onset of hands-and-knees crawling (e.g., Adolph, 1997; Adolph et al., 1998), influence infants’ postural responsiveness to PLOF? To our knowledge, no studies have examined the visual input received by belly crawlers relative to hands-and-knees crawlers and/or walkers. However, if the differentiation of PLOF is driven largely by the need to control balance more effectively and efficiently during hands-and-knees crawling, would belly crawlers have any need to differentiate and functionalize PLOF for balance control during locomotion? Presumably not, because the demands on balance control during locomotion are almost non-existent for the belly crawler. We set out to test this notion in the current experiment by comparing the postural responsiveness to side-wall/ceiling movement in a moving room of three groups of same-aged infants who were classified as either pre-crawlers, belly crawlers, or hands-and-knees crawlers. We predicted that hands-and-knees crawlers would be significantly more responsive to PLOF from side-wall/ceiling movement that specified forward motion than the pre-crawlers and belly crawlers. Thus, our experiment set out to probe an explanation for the process by which a robust developmental change in visual proprioception that occurs after the onset of hands-and-knees crawling takes place. The study is important not only because it involves a rare test of developmental process but also because it tests a basic perceptual skill, visual proprioception, that is crucial for the control and development of posture and movement and that serves as a component in more complex perceptual and spatial-cognitive skills that emerge later in development.

## Materials and Methods

### Participants

Sixty-three infants between 8 and 9 months of age were originally recruited to participate in the study. Locomotor status was first provided by parents during recruitment and then confirmed by a laboratory assessment. The original sample comprised 19 pre-crawlers, 21 belly crawlers, and 23 hands-and-knees crawlers. After confirmation by the locomotor assessment, the final sample consisted of 49 healthy, full-term infants: 19 pre-crawlers (nine female, mean age = 8.43, SD = 0.34), 15 belly crawlers (seven female, mean age = 8.58, SD = 0.27) and 15 hands-and-knees crawlers (eight female, mean age = 8.63, SD = 0.33). The hands-and-knees crawling group was considerably smaller than the original sample because we also removed infants whose parents indicated that their child had belly-crawled before crawling on hands-and-knees. The three groups of infants did not differ significantly in age, *F*(2,46) = 1.75, *p* = 0.19, and belly crawlers and hand-and-knees crawlers did not differ significantly in length of crawling experience *t*(28) = 0.76, *p* = 0.46. For the belly crawlers, the mean crawling experience was 4.84 (SD = 3.4) weeks, and for the hands-and-knees crawlers, it was 5.71 (SD = 2.56) weeks. Parents reported their infants’ ethnicity as 31 Caucasian (63.27%), 2 Asian (4.08%), 1 Hispanic (2.04%), 1 African American (2.04%), 3 Native American (6.12%), 4 Asian/Caucasian (8.16%), 6 Latino/Caucasian (12.25%), and 1 other (3 ethnicities or more, 2.04%). Parent education background included 1 below high school (2.04%), 3 high school (6.12%), 7 some college (14.29%), 13 college (26.53%), 20 master’s degree (40.82%), and 5 PhD or equivalent (10.20%). There was no difference among the three locomotor groups in the distribution of ethnicity background (*χ*^2^ = 12.13, *p* = 0.60) or parents’ education (*χ*^2^ = 13.71, *p* = 0.19). Families were representative of the diverse ethnic populations of the San Francisco Bay Area. Information on family income was not collected. The experiment was carried out in accordance with the U.S. Government’s federal regulations for the protection of human subjects and was approved by the Committee for Protection of Human Subjects at the University of California, Berkeley. All parents provided written informed consent before their infants participated in the study in accordance with the declaration of Helsinki.

### Task and Apparatus

#### The Locomotor Assessment

Upon arrival and following the provision of informed consent by the parent, each infant was given a locomotor assessment to confirm their locomotor status. Each infant had up to three trials and 2 min per trial to cross a distance of 2.5 m to the parent. Belly crawling was operationalized as prone progression with the belly in constant contact with the floor and hands-and-knees crawling was operationalized as prone progression using hands and knees with the belly supported above the ground. The infants had to demonstrate that they could cross to the parent on one of the three trials to be classified as a crawler. If the infant made no movement toward the parent on each of the three trials, the infant was classified as a pre-crawler. These definitions were based on previous investigations involving infant locomotor development (Higgins et al., 1996; Uchiyama et al., 2008; Dahl et al., 2013). As noted above, the final sample consisted of only those infants for whom the results of the locomotor assessment confirmed the infant’s locomotor status as reported by the parents. In addition, as noted above, the sample of hands-and-knees crawlers was comprised of infants who had no prior belly crawling based on parental report. Parents also indicated the date on which their infant started to belly-crawl or crawl on hands-and-knees so that crawling experience could be determined. These dates were gathered *via* a motor development questionnaire the parent filled out during the laboratory visit. The parents were encouraged to bring a baby book or diary with them to the laboratory visit and asked to estimate the closest possible date of the onset of specific motor skills with help from the above-mentioned records. The motor development questionnaire provided the parent with a brief and clear description of each skill along with an illustration. These types of retrospective questionnaires have been shown to provide valid and reliable estimates of the ages at which specific infant motor skills were acquired (e.g., Libertus and Landa, 2013).

#### The Moving Room Apparatus

The moving room was a 1.2 m × 1.2 m × 2.1 m (height × width × length) rectangular enclosure with the back wall removed. The interior walls and ceiling of the room were covered with polka-dotted fabric; the stationary floor of the room was covered with white padded fabric. The room was illuminated with two light tubes located at the top and bottom of the front wall facing a plastic infant bicycle seat. A box located in a recess in the front wall allowed the experimenter to show various toys to attract the infant’s attention during testing. At its farthest distance, the front wall was 95.5 cm from the infant, subtending a visual angle of 50° × 50°; at its nearest distance, the front wall was 62.5 cm from the infant, subtending a visual angle of 63° × 63°.

The walls of the room could be moved because of wheels underneath the side walls. The front wall could be moved independently or in conjunction with the side walls and ceiling to create different patterns of optic flow. Moving the front and side walls and ceiling together presented optic flow to the entire field of view. Moving only the side walls and ceiling presented optic flow to the peripheral field of view. The side walls and the front wall were attached to potentiometers that measured any motion of the room in either direction. Outputs were digitally sampled from the potentiometers at a rate of 50 Hz and transmitted to a desktop computer.

Any motions made by the infant were measured by voltage offsets from four pressure sensitive transducers located at the corners of a force plate under the plastic chair on which the infant was seated. Postural adjustments made by the infant changed the distribution of the forces on the pressure transducers altering the pattern of voltage offsets. The voltage offset patterns reflecting infant motion were also sampled at 50 Hz and time-locked with the samples of room motion. One high-resolution video camcorder, facing the infant, captured the face and whole body of the infant as well as a partial view of the side walls.

### Procedures

Following the locomotor assessment, the infant was placed in the infant seat inside the moving room and secured with a safety belt. One experimenter monitored the infant through a video screen and controlled the data recording for each trial. A second experimenter stood behind the front wall to capture the infant’s attention. Each trial began once the infant was still and the experimenter had gained the infant’s attention toward the front wall window. The second experimenter then signaled a third experimenter to move the walls of the moving room 35.5 cm in 2 s, alternating fore to aft (hereafter forward) and aft to fore (hereafter backward) movement for each successive trial. If the infant looked away from the front wall or held the sides of the chair prior to room movement, the trial was repeated. All infants completed 12 experimental trials plus two pseudomovement trials (one prior to the first experimental trial and one after the last trial). For the pseudomovement trials, there was no wall movement while the infant’s natural movements were captured for 2 s to get a measure of the infant’s baseline postural sway. Half the experimental trials involved exposure to whole room movement, and half involved only side-wall movement. Half of these trials involved movement of the walls toward the infant (forward) and half involved movement of the walls away from the infant (backward). The presentation order of whole room and side-wall conditions in combination with movement direction (forward and backward) was counterbalanced.

Cross-correlation between the wall movement and the infant’s postural sway was computed by a custom-written MATLAB script. A maximum cross-correlation value during the first 1.5 s of wall movement was recorded as the XMAX score. The average XMAX score of the three trials in the side-wall forward movement condition was used as an index of the infants’ responsiveness to PLOF, with a higher value indicating a greater degree of responsiveness. The average XMAX score for the three trials in the whole room forward movement condition was used as the index of infants’ responsiveness to global optic flow. The pseudomovement trials revealed that a XMAX value of 0.10 (SD = 0.11) was expectable even when the walls of the room did not move relative to the infant. In short, this means that we cannot establish a true baseline XMAX value when the baby and the walls move randomly, though it would clearly be higher than 0.10. In addition, it is important to note that we are using a maximum value of the cross correlation rather than a measure of the central tendency of the cross correlation distribution and so the baseline value is likely to be even further from 0.1.

## Results

### Primary Analyses

As we had very specific predictions, we used planned comparisons to compare the hands-and-knees crawling group to the other two groups. The first comparison showed that the hands-and-knees crawlers (XMAX = 0.55) were significantly more responsive to PLOF specifying forward motion than the pre-crawlers (XMAX = 0.39), *t*(46) = 2.44, *p* < 0.05, Cohen’s *d* = 0.84. The second test showed that the hands-and-knees crawlers were significantly more responsive to PLOF specifying forward motion than belly crawlers (XMAX = 0.41), *t*(46) = 2.14, *p* < 0.05 Cohen’s *d* = 0.78.

### Secondary Analyses

**Figure 1:**
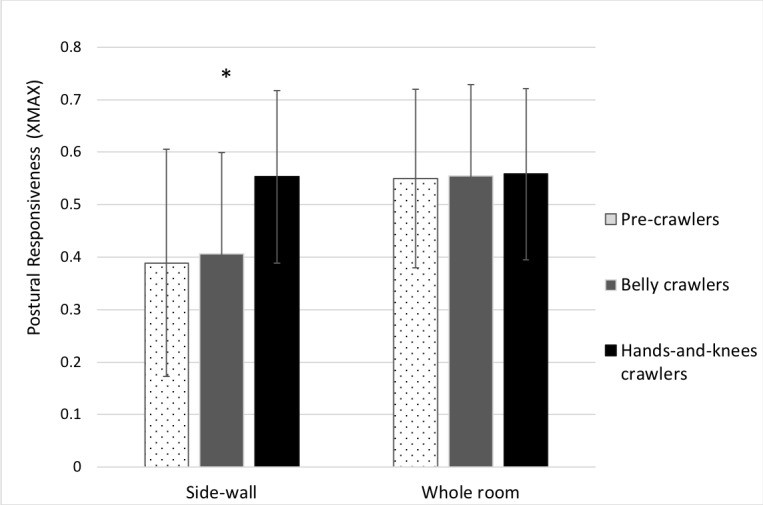
Infant postural responsiveness (XMAX) in the whole-room forward movement condition (global optic flow) and side-wall forward movement condition (peripheral lamellar optic flow). Error bar indicates +/− standard error from group means. ^*^indicates *p* < 0.05.

To confirm that the differences between the groups in their responsiveness to PLOF specifying forward motion were not attributable to the inability of pre-crawlers and belly crawlers to organize a postural response to imposed optic flow from the moving room, we ran an ANOVA on the XMAX values in the global optic flow condition in which the whole room moved toward the infants. The XMAX values for the pre-crawlers, belly crawlers, and hands-and-knees crawlers were 0.55, 0.56, and 0.56 respectively. These values were not significantly different from each other, *F*(2, 46) = 0.1, *p* = 0.99. The data for the side-wall forward (PLOF) trials and the whole room forward trials are presented in Figure 1.

## Discussion

Consistent with prior research, the findings revealed that infants with hands-and-knees crawling experience were significantly more responsive to PLOF than pre-crawlers (e.g., Higgins et al., 1996; Uchiyama et al., 2008). Moreover, consistent with our hypothesis, the hands-and-knees crawlers were significantly more responsive to PLOF than the belly crawlers, whose XMAX scores were virtually identical to those of the pre-crawlers. These findings are also consistent with prior research demonstrating an increased reliance on vision during the early acquisition of skills that involve new balance strategies (Gibson and Schmuckler, 1989; Foster et al., 1996). Together, the findings suggest that belly crawling and hands-and-knees crawling place different demands on the infant with regard to how vision is used to control balance during locomotion.

What, then, facilitates the functionalization of PLOF for the control of postural sway in the hands-and-knees crawler? We speculate that balance is a critical contributor. Effective locomotion requires a trade-off between stability and mobility. The least stable gaits are the most mobile and vice versa. For example, as the size of the base of support constricts from belly crawling to hands-and-knees crawling to walking and then to running (which is characterized by brief periods of support on a single limb interspersed with periods of flight), the balance demands become exponentially more challenging. Hands-and-knees crawling appears to be a stable gait; however, like walking, it can be described as controlled falling. The body’s center of mass is shifted forward and must be caught by the diagonally coupled hand and knee during each “step.” While in motion, the body is only supported by two diagonal limbs, whereas the other diagonal pair of limbs swing forward in preparation for the next contact with the surface. Stability in the fore-aft and medio-lateral directions is tenuous, particularly for the nascent crawler. The demands on balance control are exacerbated because the infant also has to steer an appropriate course and continuously monitor the surface of support during locomotion to a destination and all of these tasks must be managed by the visual system.

One way to effectively handle the competing demands on visual attention is to shift the visual control of balance to spatially delimited patterns of optic flow in the periphery, thus opening and freeing the central field of view to regulate steering and surface monitoring and potentially additional tasks. Note, it is also likely that somatosensory and vestibular information become more prominent in the control of crawling as proficiency increases; however, given that visual inputs tend to dominate these other inputs (e.g., Lishman and Lee, 1973; Lee and Aronson, 1974), the demands on visual attention are likely to be substantial. The process of differentiating radial and lamellar optic flow from patterns of global optic flow during locomotion should be facilitated because the head and eyes are typically oriented in the direction of travel and so the central field of view is consistently exposed to radial optic flow and the periphery is consistently exposed to lamellar optic flow. The press to differentiate lamellar optic flow and map it onto postural control strategies is far less intense for the infant who belly-crawls because the demands on balance are negligible when the infant’s torso and limbs are in constant contact with the surface of support. The negligible balance demands are a plausible reason why belly crawling does not facilitate the development of responsiveness to PLOF in the same way as hands-and-knees crawling.

Despite the logic behind the explanation we have provided for the current differences between hands-and-knees crawlers and belly crawlers, it is important to highlight that balance demands are not the only factor that separates these two groups of infants. For example, Kermoian and Campos (1988) have pointed out that belly crawling is a more effortful and less efficient form of crawling than hands-and-knees crawling, and belly crawlers devote more attention to organizing prone progression than hands-and-knees crawlers, thus limiting the attentional capacity they have available to deploy to the environment during locomotion. Kermoian and Campos argued that differences in the deployment of attention explained why hands-and-knees crawlers in one of their experiments demonstrated significantly higher performance than belly crawlers on a series of spatial search tasks. The belly crawlers performed like pre-crawling infants regardless of how much locomotor experience they had acquired. It is possible therefore that the differences between the belly crawlers and hands-and-knees crawlers in response to PLOF in the current experiment occurred because the belly crawlers have limited attentional capacity to deploy to the environment during locomotion and are thus less likely to notice the different patterns of optic flow and to differentiate them. In other words, the education of attention to meaningful information available during locomotion may be compromised in the belly crawler because of the effort and attention required to organize prone progression. Without an assessment of deployment of attention during locomotion, this argument is clearly speculative; however, it warrants consideration given the previous findings reported by Kermoian and Campos (1988). It is also important to bear in mind that the various psychological consequences that follow experience with locomotion are likely to be recruited through different processes and so belly-crawling and hands-and-knees crawling experience might have effects on spatial search and visual proprioception that are quite distinct.

Our argument that balance demands play a key role in functionalizing PLOF for postural control must also account for prior research showing that infants with locomotor experience provided by a wheeled walker or a powered-mobility-device behave like hands-and-knees crawlers when tested for responsiveness to side-wall movement in the moving room (Higgins et al., 1996; Uchiyama et al., 2008; Dahl et al., 2013). In both of these assistive locomotion devices, infants are provided with considerable postural support and so the demands on balance control are minimal, though not zero. How then does this type of locomotor experience lead to the functionalization of PLOF? One speculation is that under some circumstances, functionalization of PLOF can simply emerge from repeated exposure to the covariation between self-movement and lamellar optic flow in the visual periphery during locomotion. Assistive locomotion devices may actually create favorable circumstances for such an emergence for two reasons. First, these devices are actively controlled by the infant and so they should facilitate deployment of attention to the environment and information pick up. Agentic exploratory activity is always controlled by some anticipation of an outcome (Gibson, 1988), and stimulation obtained *via* active movement is considered more meaningful than stimulation imposed on the individual (Gibson, 1966). Second, the assistive devices minimize the attentional resources that must be devoted to controlling locomotion and thus maximize the attentional resources available for picking up meaningful information from the environment. Thus, the combination of heightened attention to the environment and attentional resource availability during locomotion may maximize the probability that the intrinsic meaning of the lamellar optic flow in the visual periphery pops out for the infant in a walker or a powered-mobility device. If true, the functionalization of PLOF for postural control might occur through alternative developmental pathways depending on whether the infant first acquires locomotion through “natural” or “artificial” means.

The current findings raise intriguing questions about developmental changes that follow experience with belly crawling and hands-and-knees crawling. For example, Dahl et al. (2013) have provided evidence that responsiveness to PLOF makes an important contribution to the emergence of wariness of heights. They hypothesized that only infants who had functionalized PLOF for balance control during locomotion would show signs of wariness on a visual cliff because only these infants would lose a source of information upon which they had come to depend when encountering a drop off. In support of the hypothesis, Dahl et al. showed a significant and positive correlation between responsiveness to PLOF and avoidance on the visual cliff as well as heart rate acceleration upon being lowered toward the deep side of the visual cliff. Infants with greater responsiveness to PLOF were more likely to avoid crossing the deep side of the cliff to the mother and demonstrated greater heart rate acceleration during lowering toward the cliff surface. In further support of the hypothesis, Anderson et al. (2018) reported that experienced hands-and-knees crawlers were significantly more likely to cross the deep side of the visual cliff when the flanks of the cliff were covered with a high-texture surface (blue and white polka dots), designed to provide PLOF, than a low-texture surface (a glossy white wall).

How would belly crawlers behave when encouraged to cross the deep side of the visual cliff to the mother or when lowered toward the deep side? Would belly crawlers behave differently than hands-and-knees crawlers when confronting a drop-off because they have not yet functionalized PLOF for balance control during crawling and so would be less concerned by the loss of PLOF at the edge of the drop-off? This question could be tested readily by examining belly crawlers on the visual cliff. However, a caveat is in order here. We have used XMAX in the current study and in previous work to establish whether infants have functionalized PLOF for postural control, but we do not assume that functionalization of PLOF is an all-or-none phenomenon and we do not know the specific threshold value of XMAX above which an infant could be said to have functionalized PLOF. Consequently, a study of belly crawlers on the visual cliff would need to assess responsiveness to PLOF in addition to avoidance on the cliff to determine the relation between these two variables. It is important to note that Dahl et al. (2013) reported that a relatively small percentage of infants with limited locomotor experience (study 1) and without locomotor experience (study 2) were already quite responsive to PLOF. Presumably, the infants were able to functionalize PLOF *via* other motoric experiences.

The process of differentiating and functionalizing PLOF for balance control likely does not end once the infant has acquired experience with hands-and-knees crawling. Is there a recalibration of visual proprioception when the child starts to stand up and walk? Do further recalibrations occur when the child acquires locomotor skills like running, hopping, and skipping that place even greater demands on the control of balance? Even within a particular mode of locomotion, it is likely that considerable refinements of visual proprioception occur just as they occur in the use of vestibular and somatosensory information. For example, Gibson and Schmuckler (1989) and Schmuckler and Gibson (1989) have shown that experienced young walkers have not completely differentiated radial and lamellar optic flow for controlling the multiple subtasks during locomotion. The young walkers experienced more staggers and falls when walking inside a moving hallway when the task involved steering around obstacles than when the pathway was uncluttered. Older children and adults appear to have refined the information available to foveal and peripheral vision during locomotion even further, evidenced by their ability to navigate through a cluttered environment without fixating objects and therefore utilizing information available in the peripheral field of view (Franchak and Adolph, 2010). Is it possible that all of the subtasks involved in controlling locomotion might ultimately be accomplished in the visual periphery, freeing the central field of view to engage in secondary tasks like monitoring other people or animals moving in the vicinity, closely inspecting a distant object during locomotion, or interacting with an object in the hand, like a cell phone?

In summary, the current findings show that different forms of prone locomotion have different effects on visual proprioception. We have argued that when balance demands during locomotion are negligible and/or considerable attentional resources are devoted to organizing prone progression, such as in belly crawling, mere exposure to radial optic flow in the central field of view and lamellar optic flow in the periphery may be insufficient to facilitate the functionalization of PLOF for balance control. Balance demands during hands-and-knees crawling may create the necessary press for functionalization to occur, though the press is ultimately a function of the need for the visual system to control multiple tasks simultaneously during locomotion. Our findings suggest that developmental changes that occur downstream from the functional utilization of PLOF for balance control, such as wariness of heights and possibly other phenomena that rely on spatial orientation, are unlikely to follow experience with belly crawling as robustly as they follow experience with hands-and-knees crawling. This hypothesis awaits experimental confirmation.

## Data Availability

The datasets generated for this study are available on request to the corresponding author.

## Ethics Statement

The experiment was carried out in accordance with the U.S. Government’s federal regulations for the protection of human subjects and was approved by the Committee for Protection of Human Subjects at the University of California, Berkeley. All parents provided written informed consent before their infants participated in the study in accordance with the declaration of Helsinki.

## Author Contributions

DA, MH, PG, IU, and JC contributed to the conception and design of the study. PG and MH collected the data, PG supervised data coding, and MH performed the statistical analyses. All authors contributed to interpretation of the analyses. DA wrote the first draft of the manuscript and all authors contributed to the revision of the manuscript. All authors edited and gave final approval for publication and were accountable for this work.

### Conflict of Interest Statement

The authors declare that the research was conducted in the absence of any commercial or financial relationships that could be construed as a potential conflict of interest.
